# Tunicamycin-Induced ER Stress is Accompanied with Oxidative Stress via Abrogation of Sulfur Amino Acids Metabolism in the Liver

**DOI:** 10.3390/ijms19124114

**Published:** 2018-12-18

**Authors:** Sou Hyun Kim, Do-young Kwon, Jae-Hwan Kwak, Seunghyun Lee, Yun-Hee Lee, Jieun Yun, Tae Gen Son, Young-Suk Jung

**Affiliations:** 1 College of Pharmacy, Pusan National University, Busan 46241, Korea; hyunie1728@naver.com (S.H.K.); tmdgus4374@naver.com (S.L.); 2 Department of Cellular and Molecular Pharmacology, University of California San Francisco, San Francisco, CA 94158-2280, USA; Doyoung.Kwon@ucsf.edu; 3 College of Pharmacy, Brain Busan 21 Plus Program, Kyungsung University, Busan 48434, Korea; jhkwak@ks.ac.kr; 4 College of Pharmacy and Research Institute of Pharmaceutical Sciences, Seoul National University, Seoul 08826, Korea; yunhee.lee@snu.ac.kr; 5 Department of Pharmaceutical Engineering, Cheongju University, Cheongju 28503, Korea; catiss@gmail.com; 6 Division for Research Center, Dongnam Institute of Radiological and Medical Science, Busan 46033, Korea; tgson@hanmail.net

**Keywords:** non-alcoholic fatty liver injury, endoplasmic reticulum stress, oxidative stress, sulfur amino acids metabolism, glutathione

## Abstract

Endoplasmic reticulum (ER) stress is involved in non-alcoholic fatty liver disease (NAFLD), but the relationship between oxidative stress, another well-known risk factor of NAFLD, and ER stress has yet to be elucidated. In this study, we treated mice with tunicamycin (TM) (2 mg/kg body weight) for 48 h to induce ER stress in the liver and examined the metabolic pathway that synthesizes the endogenous antioxidant, glutathione (GSH). Tunicamycin (TM) treatment significantly increased mRNA levels of CHOP and GRP78, and induced lipid accumulation in the liver. Lipid peroxidation in the liver tissue also increased from TM treatment (CON vs. TM; 3.0 ± 1.8 vs. 11.1 ± 0.8 nmol MDA/g liver, *p* < 0.001), which reflects an imbalance between the generation of reactive substances and antioxidant capacity. To examine the involvement of GSH synthetic pathway, we determined the metabolomic changes of sulfur amino acids in the liver. TM significantly decreased hepatic S-adenosylmethionine concentration in the methionine cycle. The levels of cysteine in the liver were increased, while taurine concentration was maintained and GSH levels profoundly decreased (CON vs. TM; 8.7 ± 1.5 vs. 5.4 ± 0.9 µmol GSH/g liver, *p* < 0.001). These results suggest that abnormal cysteine metabolism by TM treatment resulted in a decrease in GSH, followed by an increase in oxidative stress in the liver. In HepG2 cells, decreased GSH levels were examined by TM treatment in a dose dependent manner. Furthermore, pretreatment with TM in HepG2 cells potentiated oxidative cell death, by exacerbating the effects of tert-butyl hydroperoxide. In conclusion, TM-induced ER stress was accompanied by oxidative stress by reducing the GSH synthesis, which made the liver more susceptible to oxidative stress.

## 1. Introduction

Non-alcoholic fatty liver disease (NAFLD) represents a spectrum of liver conditions from simple lipid accumulation to steatohepatitis, liver fibrosis, and hepatocellular carcinoma [1,2,3]. Excessive lipid accumulation in the liver, namely fatty liver or hepatic steatosis, is caused by abnormal lipid metabolism, and subsequent progression is exasperated by inflammatory cytokines and oxidative stress [4,5,6].

The endoplasmic reticulum (ER) is the major site of lipid metabolism in hepatocytes, and disruption of homeostasis in the ER, referred to as ER stress, plays a critical role in the progression of fatty liver and hepatic lipid accumulation [7,8]. Although ER stress is considered a major contributor to the induction of fatty liver disease, accumulating studies suggest that oxidative stress is another critical factor in the development of NAFLD [9,10,11]. Oxidative stress is a primary cause of fat accumulation in the liver and is involved in the development of fibrosis in NAFLD patients [12,13,14]. In a liver with accumulated fat, increased production of reactive oxygen species (ROS) causes lipid peroxidation, followed by inflammation and fibrogenesis [7,15]. Therefore, it is currently accepted that these two cellular stresses, ER-stress and oxidative stress, are closely linked in the maintenance of hepatic homeostasis [16]. However, the functional interactions between ER stress and oxidative stress in the development and progression of NAFLD remain unclear.

Several reports demonstrated that tunicamycin (TM) could efficiently induce ER stress in the liver of mice [17,18] and it has been used as an experimental model to study the effect and mechanism by which ER stress regulates lipid metabolism [19,20,21,22,23], energy homeostasis [24,25], inflammation [26,27], and fibrogenesis [28] in the liver. The purpose of this study was to investigate how ER stress-induced hepatic lipid accumulation affects the redox balance in the liver. Specifically, this study was focused on the changes in the metabolic pathway of sulfur-containing substances by acute treatment of the ER stressor, TM. The metabolism of sulfur-containing substances is connected with the biosynthesis of glutathione (GSH), a potent antioxidant that scavenges reactive metabolites, and the homeostasis of this metabolic process plays a critical role in the susceptibility to toxic oxidants [29,30,31]. Here, we demonstrated that exposure to TM-induced abnormal changes in the metabolism of sulfur-containing substances weakened the antioxidant capacity via the inhibition of de novo GSH synthesis in the liver, specifically.

## 2. Results

### 2.1. ER Stress-Mediated Lipid Accumulation in the Liver of Tunicamycin (TM)-Treated Mice

**Figure 1:**
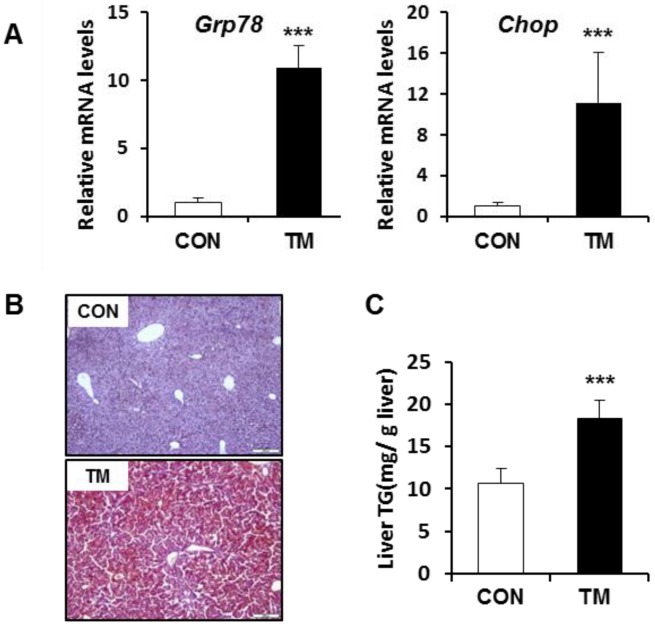
Endoplasmic reticulum (ER) stress-mediated lipid accumulation in the liver of tunicamycin (TM)-treated mice. (**A**) mRNA levels of glucose-regulated protein 78 (Grp78) and CCAAT/enhancer-binding protein homologous protein (CHOP), and (**B**) Oil Red O staining in the liver (20× magnification). (**C**) Levels of triglyceride in the homogenates of the liver. Each value represents the mean ± SD. *** Statistically significant difference between control (white) and TM-treated mice (black) at *p* < 0.001 (Student’s *t*-test).

The expression of binding immunoglobulin protein/glucose-regulated protein 78 kDa (Bip/Grp78), as a representative ER chaperone, was examined in the liver 48 h after treatment to monitor the TM-induced ER stress response. As shown in Figure 1A, BIP/GRP78 mRNA expression was significantly elevated in the liver of TM-treated mice. Simultaneously, the mRNA expression of CCAAT/enhancer-binding protein homologous protein (CHOP), one of the ER stress-response genes, was also significantly enhanced by tunicamycin (TM) treatment. In a line with previous studies, TM treatment induced lipid accumulation in the liver, as evidenced by Oil Red O lipid staining of liver tissue (Figure 1B) and biochemical analysis of hepatic lipid levels (Figure 1C).

### 2.2. Changes in mRNA Level Related with Inflammatory Response and Fibrogenesis in the Liver of TM-Treated Mice

**Figure 2:**
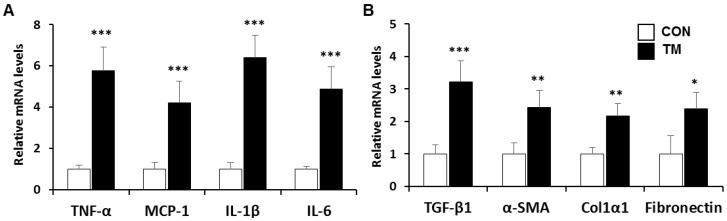
mRNA expression associated with (**A**) inflammatory response and (**B**) fibrogenesis in the liver of TM-treated mice. Each value represents the mean ± SD. *, **, and *** Statistically significant difference between control (white) and TM-treated mice (black) at *p* < 0.05, < 0.01, and < 0.001, respectively (Student’s *t*-test).

In the animal model and human patients of NAFLD, ER stress was accompanied by hepatic steatosis, inflammation, as well as fibrosis lesion in non-alcoholic steatohepatitis (NASH) [27,28]. To examine whether TM-induced ER stress has an impact on the inflammation and fibrosis, we determined the levels of mRNA associated with inflammatory response and fibrogenesis. The mRNA levels of inflammatory genes, such as tumor necrosis factor alpha (TNF-α), monocyte chemoattractant protein-1 (MCP-1), interleukin 1 beta (IL-1β), and interleukin 6 (IL-6) were significantly increased to 5.7-, 4.2-, 6.4-, and 4.9-fold, respectively, in the liver of TM-treated mice (Figure 2A). Fibrosis markers, such as transforming growth factor beta 1 (TGF-β1), alpha smooth muscle actin (α-SMA), alpha-1 type I collagen (Col1α1), and fibronectin also increased significantly, but the increased range was smaller than that of inflammatory response genes (Figure 2B). These results suggest that the acute challenge of TM induces similar stage to NASH that shows lipid accumulation, inflammation, weak fibrosis.

### 2.3. Increased Hepatotoxicity Accompanied with Oxidative Stress due to TM

**Figure 3:**
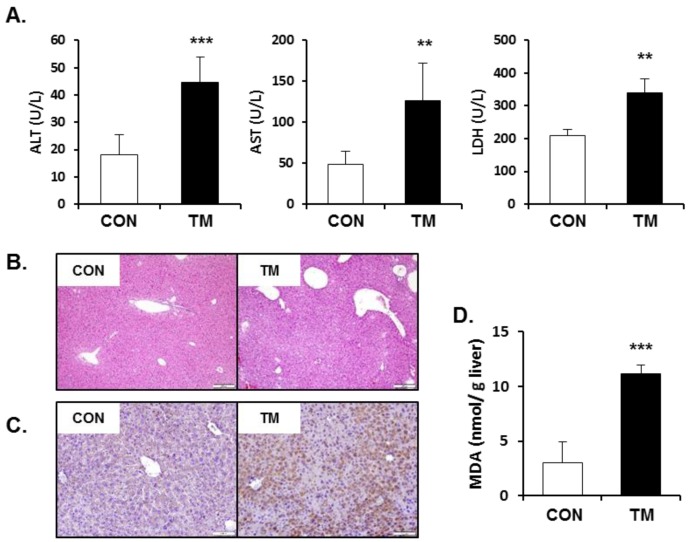
Increased hepatotoxicity accompanied with oxidative stress by tunicamycin (TM) treatment. (**A**) Serum activities of alanine aminotransferase (ALT), aspartate aminotransferase (AST), and lactate dehydrogenase (LDH) in TM-treated mice (2 mg/kg body weight). (**B**) Hematoxylin and eosin (H&E) staining of liver tissues to examine histopathological changes (20× magnification). (**C**) Immunohistochemistry of hepatic 4-hydroxynonenal (4-HNE; 20× magnification) and (**D**) malondialdehyde (MDA) levels in the liver tissues to evaluate lipid peroxidation. Each value represents the mean ± SD. ** and *** Statistically significant difference between control (white) and TM-treated mice (black) at *p* < 0.01 and < 0.001, respectively (Student’s *t*-test).

The serum concentrations of alanine aminotransferase (ALT), aspartate aminotransferase (AST), and lactate dehydrogenase (LDH) in TM-treated mice (2 mg/kg body weight) significantly increased compared to control mice 48 h after treatment (Figure 3A). Hematoxylin and eosin (H&E) staining of liver tissues to examine histopathological changes did not show clear differences between the control and TM-treated mice (Figure 3B), suggesting that a TM concentration of 2 mg/kg induced only low-grade toxicity in the liver, as evidenced by the increased biochemical indices. Recently, it has been suggested that the highly interrelated processes between ER stress and redox imbalance are involved in the pathogenesis of human disease [32]. Interestingly, hepatic 4-hydroxynonenal (4-HNE) staining (Figure 3C) and the levels of malondialdehyde (MDA) (Figure 3D) in the liver, which evaluated lipid peroxidation, were significantly enhanced by the treatment of TM. These results suggest that liver injury by TM might be involved in both ER stress and oxidative stress.

### 2.4. Changes in Hepatic Metabolism of Sulfur Containing Substances due to TM Treatment

**Figure 4:**
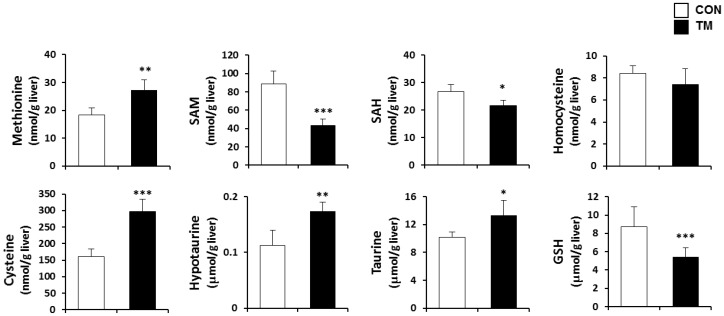
Changes in the hepatic sulfur containing substances by the TM treatment. Hepatic concentration of methionine, S-adenosylmethionine (SAM), S-adenosylhomocysteine (SAH), homocysteine, cysteine, hypotaurine, taurine, and glutathione (GSH) were measured in TM-treated mice (2 mg/kg body weight) using the analytic system of high-performance liquid chromatography (HPLC). Each value represents the mean ± SD. *, **, and *** Statistically significant difference between control (white) and TM-treated mice (black) at *p* < 0.05, < 0.01, and < 0.001, respectively (Student’s *t*-test).

To determine the effect of TM treatment on the GSH synthetic pathway in the liver, the concentration of sulfur containing substances and their metabolites were monitored in control and TM-treated mice. Hepatic methionine, cysteine, hypotaurine, and taurine levels were 150%, 200%, 130%, and 120% of control values in TM-treated mice, respectively (Figure 4). Whereas the hepatic concentration of S-adenosylmethionine (SAM), S-adenosylhomocysteine (SAH), and GSH were significantly decreased to 50%, 80%, and 50% of control values in TM-treated mice, respectively. There were no significant differences in hepatic homocysteine levels.

**Figure 5:**
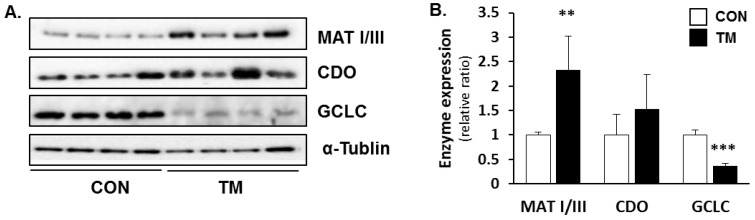
Changes in protein expression of enzymes involved in transsulfuration pathway after TM treatment. (**A**) Protein expression of hepatic methionine adenosyltransferase (MAT) I/III, CDO, and the catalytic subunit of GCL (GCLC) were determined by immunoblot analysis in TM-treated mice (2 mg/kg body weight), and (**B**) were quantified densitometrically. Each value represents the mean ± SD. ** and *** Statistically significant difference between control (white) and TM-treated mice (black) at *p* < 0.01 and < 0.001, respectively (Student’s *t*-test).

The metabolic changes of sulfur-containing substances were determined by measuring the protein levels of the hepatic enzymes involved in this metabolism using western blotting. The protein expression of methionine adenosyltransferase (MAT) I/III, which converts methionine to SAM using ATP, was significantly increased, and the protein expression of CDO, a rate-liming step of the taurine synthesis, also increased, but the increase was not significant (Figure 5A,B). The protein levels of GCLC, the catalytic subunit of GCL, which is involved in de novo synthesis of GSH, were dramatically decreased in the liver of TM-treated mice, reflecting the low concentrations of GSH (Figure 5A,B).

### 2.5. Decreased Levels of Cellular GSH by TM Treatment in a Dose-Dependent Manner and Potentiation of t-BHP Induced Cell Death by Pre-Exposure of TM in HepG2 Liver Cells

**Figure 6:**
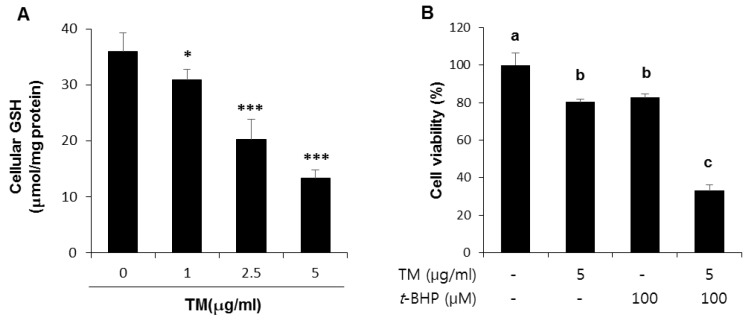
Effect of TM treatment on the cellular level of GSH, and potentiation of oxidative cell death by the pre-exposure of TM in HepG2 liver cells. (**A**) Cellular levels of GSH at 24 h after treatment with the indicated concentration of TM. (**B**) Cells were exposed to 5 µg of TM for 24 h before treatment with 300 µM of tert-butyl hydroperoxide (*t*-BHP) for 6 h, followed by an MTT assay. Each value represents the mean ± SD. * and *** Statistically significant difference from vehicle treated cells indicated by “TM 0” at *p* < 0.05 and < 0.001, respectively (Student’s *t*-test). Values with different letters are significantly different from one another (ANOVA followed by Newman-Keuls multiple range test *p* < 0.05).

Our animal study suggests that TN treatment reduced GSH synthesis and weakened the antioxidant capability of the liver. Therefore, it is hypothesized that pre-exposure to TM can make cells sensitive to the second oxidant attack, which would potentiate the harmful effects of oxidative stress. Cellular GSH levels were decreased in a dose-dependent manner at 24 h after TM treatment in HepG2 liver cells (Figure 6A). To investigate whether the decrease in cellular GSH due to TM treatment affected cell viability in the presence of exogenous oxidative stress, TM-treated HepG2 cells were subsequently exposed to *t*-BHP, which produces ROS, and cell viability was examined 6 h post-treatment (Figure 6B). As expected, cells that were pre-exposed to TM were more sensitive to *t*-BHP treatment, leading to 70% cell death from 20% cell death induced by TM alone or *t*-BHP alone.

### 2.6. Involvement of Mitochondrial Dysfunction in the Enhancement of t-BHP-Induced Mitochondrial Dysfunction in the TM Pre-Exposed HepG2 Liver Cells

**Figure 7:**
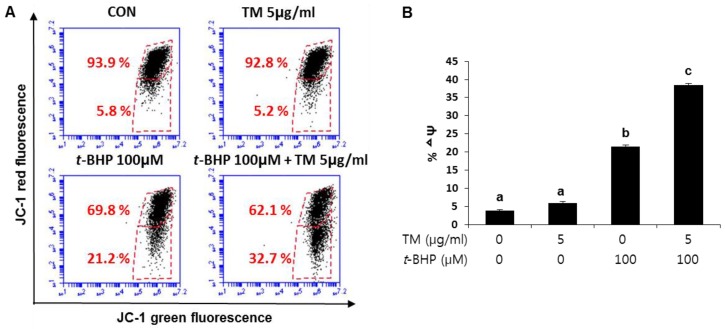
Enhancement of *t*-BHP-induced mitochondrial dysfunction by the pre-exposure of TM in HepG2 liver cells. (**A**) Cells were exposed to 5 µg of TM for 24 h before treatment with 100 µM of *t*-BHP for 3 h to accomplish FACS analysis of JC-1 staining. (**B**) Quantification of the mitochondrial membrane potential (∆ψ) was represented in the three independent experiments. Each value represents the mean ± SD. Values with different letters are significantly different from one another (ANOVA followed by Newman-Keuls multiple range test *p* < 0.05).

ER stress can cause mitochondrial dysfunction and increase mitochondrial ROS generation, leading to oxidative stress. The mitochondrial membrane potential (∆ψ) is a sensitive indicator for the energetic state of the mitochondria, and it was measured to assess mitochondrial function. JC-1, a dye that selectively enters the mitochondria, reversibly changes color as the ∆ψ changes. As shown in Figure 7, treatment with TM alone for 24 h did not induce a change in ∆ψ compared to control cells. *t*-BHP treatment alone significantly increased depolarized cells from 5.8% in control cells to 21.2% of *t*-BHP treated cells. Whereas exposure to *t*-BHP after TM treatment significantly increased the depolarized cell population to 32.7% of total cells, suggesting that pretreatment with TM made the mitochondria more vulnerable to additional stress, followed by aggravating mitochondrial dysfunction.

### 2.7. Enhancement of t-BHP-Induced Apoptotic Cell Death by Pre-Exposure of TM in HepG2 Liver Cells

**Figure 8:**
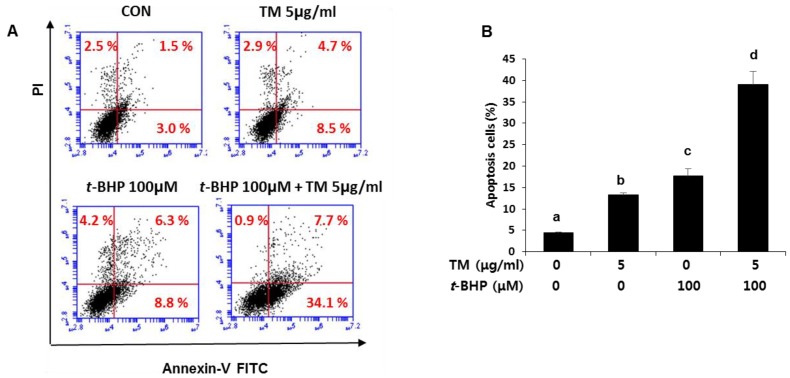
Potentiation of *t*-BHP-induced apoptotic cell death by the pre-exposure of TM in HepG2 liver cells. (**A**) Cells were exposed to 5 µg of TM for 24 h before treatment with 100 µM of *t*-BHP for 6 h. Fluorescence-activated cell sorting (FACS) analysis of propidium iodide uptake and annexin V binding in non-permeabilized cells (Lower-left, live cells; Lower-right, early apoptotic cells; upper-right, late apoptotic cells). (**B**) Quantification of apoptotic cells was represented in the three independent experiments. Values with different letters are significantly different from one another (ANOVA followed by Newman-Keuls multiple range test *p* < 0.05).

The effect of TM and *t*-BHP treatments on apoptosis was determined by flow cytometry analysis using Annexin V and PI staining. As shown in Figure 8A,B, an increase in apoptosis (sum of upper and lower right quadrants) was clearly observed in cells treated with *t*-BHP after TM exposure (apoptosis in 41% of total cells) compared with TM alone (apoptosis in 13% of total cells) or *t*-BHP alone (apoptosis in 15% of total cells).

## 3. Discussion

NAFLD is considered one of the main causes of chronic liver disease worldwide [1]. In many patients, NAFLD does not progress further than simple hepatic steatosis; however, there is a significant proportion of patients with NASH, a severe form of NAFLD, which can lead to cirrhosis, hepatocellular carcinoma (HCC), and liver disease-related death [2,3]. The ‘two-hit hypothesis’ was a widespread theory that explains the pathogenesis and progression of NAFLD [33]. This theory suggests that in the steatosis alone, due to a high fat diet or alcohol intake, a second hit from other factors, such as oxidant insult was required for the development of NASH [9]. However, it is not appropriate to describe the complex molecular and metabolic changes that occur in NAFLD. Accumulating studies have shown that multiple sequential or parallel pathways are involved in disease progression, and the crosstalk between the multiple pathways still remains to be explored [34,35,36,37]. In this study, we observed that TM-induced ER stress in the liver was accompanied with multiple changes, such as a decrease in GSH concentration, an increase in inflammatory response and fibrogenesis, which could contribute to the progression of the disease. Among them, we have further investigated the effect of TM-induced ER stress on the GSH synthetic pathway leading to a redox imbalance.

In the present study, it is notable that the liver of TM-treated mice showed a significant increase in oxidative stress, as evidenced by higher levels of 4-HNE and MDA compared to the liver of vehicle-treated mice. Oxidative stress is considered a key role in the progression of NAFLD from disease initiation to NASH, even if the cause and effect relationship between oxidative stress and the pathogenesis of NAFLD has not yet been clearly elucidated [38]. Oxidative stress is caused by the elevated formation of ROS and the suppression of the endogenous antioxidant capacity, which initiates lipid peroxidation. Subsequently, highly reactive aldehyde components, such as 4-HNE and MDA, cause intracellular damage [16]. In fact, markers reflecting lipid peroxidation have been observed in NAFLD/NASH patients, and the concentration of these markers are proportionally related to the severity of liver disease [39,40,41,42].

**Figure 9:**
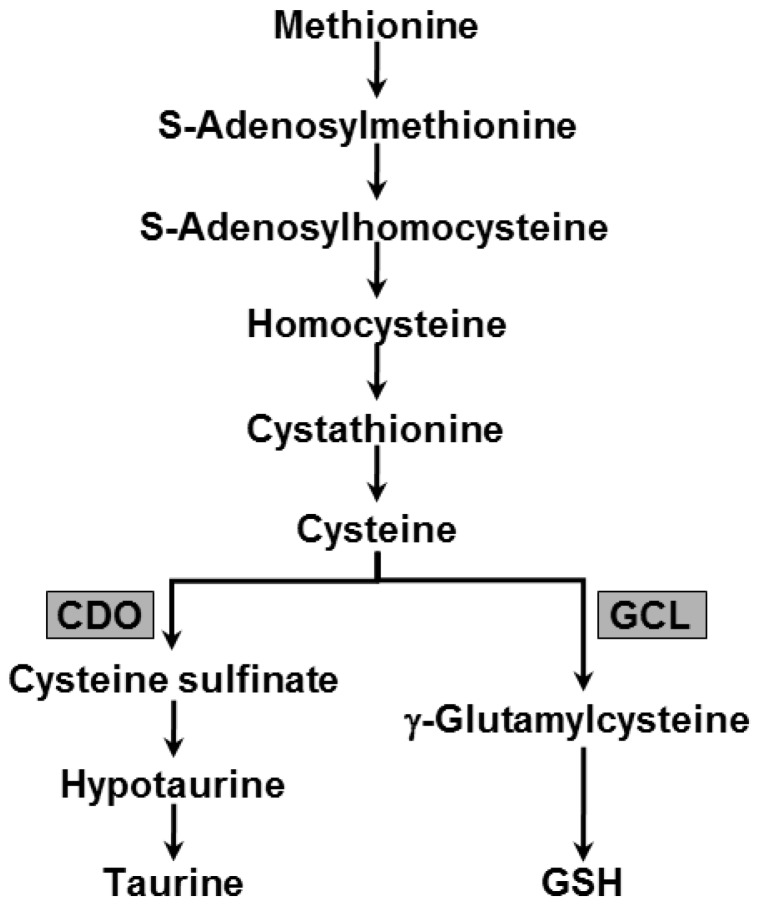
Transsulfuration pathway in the liver. CDO, cysteinedioxygenase; GCL, γ-glutamylcysteineligase; GSH, glutathione.

Oxidative stress by TM treatment seems to be caused by the changes in the synthetic pathway of GSH, which is the most abundant non-protein thiol available to defend reactive metabolites in all mammalian tissues. GSH is a tripeptide composed of glutamate, cysteine, and glycine, with the thiol group in cysteine, in particular, providing the antioxidant ability of GSH. GSH is synthesized from the essential amino acid, methionine, through a transsulfuration process, and the synthesis of GSH in the liver is mainly regulated by two factors, the availability of cysteine and the activity of GCL (Figure 9) [30,43]. In this study, protein expression of GCLC was dramatically inhibited by TM, which resulted in a decrease in GSH synthesis in the liver of TM-treated mice. In contrast, CDO expression was slightly increased with elevation of both hypotaurine and taurine concentrations. The results suggest that cysteine catabolism into taurine was favored over GSH production, which may have intensified the TM-induced GSH depletion in the liver. Moreover, a chemical oxidant, tert-butyl hydroperoxide (*t*-BHP), induced potent cell death in ER stress-induced liver cells. These findings suggest the importance of sulfur amino acid metabolism in the ER stress-dependent fatty liver.

In conclusion, TM-induced ER stress was accompanied by oxidative stress, due to a reduction in GSH synthesis, which made the fatty liver of TM-treated mice more susceptible to additional oxidative stress. Thus, this study supports the possibility that supplementation with antioxidants may help to inhibit the progression of NAFLD.

## 4. Materials and Methods

### 4.1. Animals Experiment

Seven-week old male DBA/2Korl mice were kindly provided by the Department of Laboratory Animal Resources in the National Institute of Food and Drug Safety Evaluation (NIFDS, Cheongju, Korea). Animals were acclimated to the temperature (22 ± 2 °C) and humidity (55 ± 5%) controlled rooms with a 12 h light/dark cycle for one week before use. Mice were treated with TM (2 mg/kg, ip) and sacrificed 48 h after treatment. The use of animals was in compliance with the guidelines established and approved by the Animal Care and Use Committee in Pusan National University (approval No. PNU-2016-1192).

### 4.2. Hematological Evaluation Indicating Hepatotoxicity

The serum activities of ATL, AST, and LDH in blood samples were examined with an Automated Chemistry Analyzer (Prestige 24I; Tokyo Boeki Medical System, Tokyo, Japan).

### 4.3. Histopathological Analysis

The liver tissues were fixed in 10% buffered neutral formalin and embedded in a low-melting-point paraffin. Tissue sections 5 µm in thickness were stained with H&E and mounted using DPX mountant, followed by microscopic examination (Olympus CX41RF; Olympus Co., Tokyo, Japan).

### 4.4. Determination of Triglyceride (TG) in the Liver

Total lipids were extracted from a homogenate prepared from 200 mg of a mouse liver in a chloroform/methanol mix (2:1, *v*/*v*). TG levels in the total lipid extract were determined enzymatically using a commercially available enzymatic kit (Sigma Chemical Co., St. Louis, MO, USA) according to the manufacturer′s protocol. In addition, to evaluate lipid staining in the liver, 5 µm-thick cross sections of the left lateral lobe of the liver were immersed in propylene glycol and stained with Oil Red O reagent.

### 4.5. Examination of Lipid Peroxidation in the Liver

A cross section of the liver was sliced at 5 µm and stained using rabbit polyclonal anti-4-HNE (Abcam, Cambridge, MA, USA) and goat anti-rabbit polyclonal secondary antibodies (Vectastain ABC IHC kit; Vector Laboratories, Burlingame, CA, USA) to evaluate 4-HNE adducts. Overall, liver lipid peroxidation was determined by a thiobarbituric acid reactive substrate (TBARS) assay as described by Volpi and Tarugi [44]. The liver lysate was mixed with 0.2% thiobarbituric acid in 2 M sodium acetate buffer containing 5% butylated hydroxytoluene. The mixtures were incubated at 95 °C for 45 min followed by centrifugation. The supernatant was injected into high performance liquid chromatography (HPLC), equipped with a fluorescence detector (FLD-3100; Thermo Scientific, Sunnyvale, CA, USA) and a 5 µm Symmetry C18 reversed phase column (4.6 mm × 150 mm; Eka Chemicals, Bohus, Sweden). The complex of MDA and thiobarbituric acid was monitored by fluorescence detection with excitation at 515 nm and emission at 553 nm.

### 4.6. Real-Time Reverse Transcription-Polymerase Chain Reaction (RT-PCR)

The total RNA was isolated from cells using the Direct-zol™ RNA kit (Zymo Research, Orange, CA, USA). The cDNA was synthesized by the iScript™ cDNA Synthesis system (Bio-Rad, Hercules, CA, USA). The reak-time RT-PCR was accomplished by using the SensiFAST SYBR qPCR mix (Bioline, London, UK) according to the manufacturer’s protocol. The relative values of gene expression were normalized to GAPDH. The primer sequences are provided in Table 1.

### 4.7. Western Blotting

Liver tissue was lysed with ice-cold PRO-PREP^TM^ protein extract solution (iNtRON; Sungnam, Gyunggi, Korea), and the concentration of the protein was determined by the BCA reagent (Thermo Scientific, Sunnyvale, CA, USA). Equal amounts of protein were separated by SDS-PAGE and transferred onto a nitrocellulose membrane (Bio-Rad, Hercules, CA, USA). The membranes were incubated with TBS-T containing 5% milk and the primary antibodies against MAT I/III, GCLC, α-tubulin (Santa Cruz Biotechnology, Santa Cruz, CA, USA), and CDO (Abcam, Cambridge, MA, USA). After washing with TBS-T, the membrane was incubated with the appropriate horseradish peroxidase-conjugated secondary antibodies. The antigen was detected using a Western Bright ECL HRP substrate kit (Advansta, Menlo Park, CA, USA).

### 4.8. Determination of Sulfur-Containing Substances

Methionine, hypotaurine, and taurine were derivatized with *ο*-phthalaldehyde/2-mercaptoethanol and quantified using an HPLC with a fluorescence detector (FLD-3100; Thermo Scientific; excitation 338 nm and emission 425 nm) [43,45]. They were separated with a Hector T-C18 column (3 µm × 4.6 mm × 100 mm; Chiral Technology Korea, Daejeon, Korea). The examination of both SAM and SAH concentrations were determined by HPLC separation with a UV detector (UltiMate™ 3000 VWD; Thermo Scientific; 254 nm). Homocysteine, cysteine, and GSH were analyzed by the SBD-F derivatization method [46] using an HPLC with a fluorescence detector (FLD-3100; Thermo Scientific; excitation 385 nm and emission 515 nm). The chromatographic separation was achieved with a Hector M-C18 column (3 µm × 4.6 mm × 150 mm: Chiral Technology Korea).

### 4.9. Cell Culture

HepG2 human liver cells (ATCC, Manassas, VA, USA) were grown in Dulbecco’s modified eagle’s medium (DMEM) with 10% fetal bovine serum (FBS), 2 mM glutamine, 100 U/mL penicillin, and 100 µg/mL streptomycin (GenDEPOT, Barker, TX, USA) at 37 °C in a humidified incubator with 5% CO_2_.

### 4.10. Determination of Cell Viability

Cell viability was determined by the MTT assay, as instructed by the manufacturer. Briefly, after incubation with MTT (0.5 mg/mL, Sigma Chemical Co.) for 4 h at 37 °C, formazan precipitates formed by mitochondrial dehydrogenases in viable cells were extracted with DMSO. The absorbance of the converted dye was measured at 540 nm using the MULTISKAN GO reader (Thermo Scientific), and the results were expressed as a percentage (%) compared to the vehicle-treated cells.

### 4.11. Analysis of Mitochondrial Membrane Potential (∆ψ)

HepG2 cells were incubated with 5 µg/mL of TM for 24 h, and later treated with 100 µM *t*-BHP for 3 h. After treatment, cells were incubated with 10 µM JC-1 in media for 30 min in the dark, and later collected by scraping. After the cells were washed in PBS, they were subjected to fluorescence-activated cell sorting (FACS) analysis. There are two excitation wavelengths, 527 nm (green) for the JC-1 monomer and 590 nm (red) for JC-1 aggregates. With normal mitochondrial function, mitochondrial membrane potential (∆ψ) is high, and the red fluorescence signal is predominant. However, when there is mitochondrial injury, mitochondrial membrane potential (∆ψ) is reduced, leading to an increase in the green fluorescence signal. Quantitation of red and green fluorescent signals reflects whether mitochondria are damaged. The change in ∆ψ was monitored by the Becton Dickinson FACSscan flow cytometer and BD FACSDiva software (BD Biosciences, San Jose, CA, USA).

### 4.12. Determination of Apoptotic Cells Using FACS Analysis

FACS analysis using Annexin V-fluorescein isothiocyanate (FITC) staining identified both apoptotic and live cells. HepG2 cells were incubated with 5 µg/mL of TM for 24 h, and the cells were later treated with 100 µM of *t*-BHP for 6 h. After harvesting, the counted cells were stained in propidium iodide and Annexin V-FITC solution (Annexin V-FITC Apoptosis Detection Kit; BD Biosciences) at room temperature for 15 min in the dark. The stained cells were analyzed by flow cytometry within 1 h. Both apoptotic and live cells were analyzed by the Becton Dickinson FACSscan flow cytometer and BD FACSDiva software (BD Biosciences).

### 4.13. Statistical Analysis

All results, expressed as mean ± SD, were analyzed by a two-tailed Student’s *t*-test or a one-way analysis of variance (ANOVA) followed by the Newman-Keuls multiple comparisons test. The acceptable level of significance was established at *p* < 0.05.

## Figures and Tables

**Table 1:** List of mouse primer used for real-time reverse transcription-polymerase chain reaction (RT-PCR).

Genes	Primer Sequences	
Grp78	F: TGGTATTCTCCGAGTGACAGC	R: AGTCTTCAATGTCCGCATCC
Chop	F: CACGCACATCCCAAAGCC	R: GGGCACTGACCACTCTGTT
TNFα	F: GGCCTCTCTACCTTGTTGCC	R: CAGCCTGGTCACCAAATCAG
CCL2	F: CCAGCAAGATGATCCCAATG	R: CTTCTTGGGGTCAGCACAGA
IL1β	F: TTCACCATGGAATCCGTGTC	R: GTCTTGGCCGAGGACTAAGG
IL6	F: TTGCCTTCTTGGGACTGATG	R: CCACGATTTCCCAGAGAACA
TGFβ1	F: GCCCTGGATACCAACTATTGC	R: TGTTGGACAGCTGCTCCACCT
αSMA	F: GGCTCTGGGCTCTGTAAGG	R: CTCTTGCTCTGGGCTTCATC
Col1a1	F: ACCTGTGTGTTCCCTACTCA	R: GACTGTTGCCTTCGCCTCTG
Fibronectin	F: ATGACGATGGGAAGACCTAC	R: GGCTGGAAAGATTACTCTCG
GAPDH	F: GTTGTCTCCTGCGACTTCA	R: GGTGGTCCAGGGTTTCTTA

## Abbreviations

NAFLD	Non-alcoholic fatty liver disease
NASH	Non-alcoholic steatohepatitis
TM	tunicamycin
ER	endoplasmic reticulum
ROS	reactive oxygen species
GSH	glutathione
*t*-BHP	tert-butyl hydroperoxide
